# The RNA-binding protein CELF4: from molecular regulatory networks to clinical translation in cross-system diseases

**DOI:** 10.3389/fmolb.2026.1883784

**Published:** 2026-07-14

**Authors:** Qingsong Wang, Wenlong Yue, Dan Lin, Xianmin Wang, Tongyong Luo, Jun Yin

**Affiliations:** 1 Department of Pediatrics, West China Hospital Sichuan University Jintang Hospital. Jintang First People’s Hospital, Chengdu, Sichuan, China; 2 Department of Neurosurgery, West China Hospital Sichuan University Jintang Hospital. Jintang First People’s Hospital, Chengdu, Sichuan, China; 3 Department of Internal Medicine, Guancang Community Health Service Center, Chengdu, Sichuan, China; 4 Pediatric Cardiology Center, Sichuan Provincial Women’s and Children’s Hospital, The Affiliated Women’s and Children’s Hospital of Chengdu Medical College, Chengdu, Sichuan, China; 5 Department of Ultrasound, West China Hospital Sichuan University Jintang Hospital. Jintang First People’s Hospital, Chengdu, Sichuan, China

**Keywords:** cardiac fibrosis, Celf4, precision medicine, RNA-binding protein, synaptic homeostasis, translational repression

## Abstract

CELF4 (CUGBP Elav-like family member 4), encoded by the human chromosome 18q12.2 locus, is an RNA-binding protein that recognizes UG-rich sequences within the 3′untranslated region (3′UTR) of target mRNAs to regulate splicing, stability, and local translation at the post-transcriptional level. Under physiological conditions, CELF4 exerts translational repression during synaptic development in the central nervous system (CNS), maintains excitatory homeostasis, and sets peripheral sensory thresholds; in cardiac fibroblasts, it is expressed at low levels and restricts baseline TGF-β signaling. In pathological states, CELF4 exhibits context-dependent bidirectional modulation: in autism spectrum disorder (ASD), major depressive disorder (MDD), epilepsy, chronic pain, and endometrial cancer, its downregulation or epigenetic silencing causes translational derepression of target mRNAs; in cardiac fibrosis, TGF-β1-induced upregulation suppresses FMO2 translation and activates the Smad2/3 pathway. Additionally, pleiotropic genetic loci near CELF4 have been linked to gut-brain axis comorbidities and obesity-hypertension syndromes. Clinically, CELF4 promoter methylation testing has entered validation trials for non-invasive endometrial cancer screening, and its haploinsufficiency has been incorporated into the genetic diagnosis of 18q12.2 microdeletion syndrome; pharmacological and gene-replacement strategies targeting CELF4 remain at the preclinical proof-of-concept stage. Here, we review the molecular regulatory networks of CELF4 and its mechanisms across multisystem diseases, discuss the current status and limitations of clinical translation, and may guide future research on diagnostic biomarkers and therapeutic strategies targeting this protein.

## 1 Biological characteristics of CELF4

### 1.1 Molecular features

**FIGURE 1:**
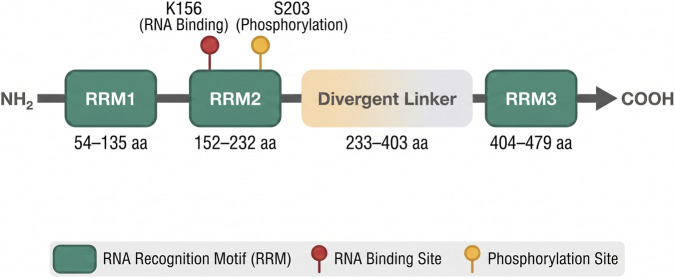
Domain architecture and key functional sites of the CELF4 protein. CELF4 is a member of the CUGBP Elav-like family, structurally characterized by two closely spaced, tandem RNA recognition motifs (RRM1 and RRM2) at the N-terminus, a central divergent domain (Inter-RRM Linker), and a single RRM (RRM3) near the C-terminus. The RRMs collectively mediate specific binding to target mRNA transcripts, whereas the central linker is highly variable and involved in alternative protein functions. Key functional residues, including the RNA-binding site (K156) and the phosphorylation site (S203), are mapped within the RRM2 domain, highlighting their critical roles in CELF4 post-transcriptional regulatory activity.

The human *CELF4* gene (CUGBP Elav-like family member 4, also known as *BRUNOL4*) maps to chromosomal region 18q12.2. Multiple transcript isoforms exist; the canonical transcript is NM_020180.4, encoding a mature protein of approximately 52 kDa (Bruel et al., 2025). The CELF4 protein is highly conserved across evolution, and its core functional domain consists of three RNA recognition motifs (RRMs): two tandem RRMs (RRM1 and RRM2) at the N-terminus and a single RRM3 at the C-terminus. Together, these domains enable CELF4 to bind target mRNAs with sequence specificity (Wagnon et al., 2012). Database analyses (Transite and ATTRACT) indicate that CELF4 preferentially recognizes UG-rich repeats in the 3′UTR of target mRNAs; its classic RNA-binding motifs have been identified as UGUGUKK or UGUGUGU (Grlickova-Duzevik et al., 2023). The subcellular distribution of CELF4 is consistent with its role in translational regulation. In central neurons, the protein is predominantly cytoplasmic and is highly enriched in the neuropil, where it co-sediments with polysomes; this spatial distribution constitutes the structural basis for its local translational repression (Wagnon et al., 2012). In non-neural tissues such as cardiac fibroblasts, CELF4 is likewise distributed mainly in the cytoplasm (Zhang et al., 2025) (Figure 1).

### 1.2 Expression regulatory mechanisms

#### 1.2.1 Developmental and tissue-specific expression

CELF4 is broadly expressed during early embryonic development, but its expression pattern contracts sharply after birth, becoming progressively restricted to the CNS (Salamon et al., 2023). In adulthood, its mRNA is enriched in the cerebral cortex, hippocampus, and cerebellum (Barone et al., 2017). In the peripheral nervous system (PNS), CELF4 is specifically expressed in small-to medium-diameter sensory neurons of the dorsal root ganglion (DRG); subpopulation analyses reveal high expression in peptidergic (CGRP+) neurons and low expression in non-peptidergic (IB4-binding) neurons (Grlickova-Duzevik et al., 2023). In the cardiovascular system, CELF4 protein is detectable only in cardiac fibroblasts and is virtually absent from cardiomyocytes, peripheral blood mononuclear cells (PBMCs), and human umbilical vein endothelial cells (HUVECs) (Zhang et al., 2025).

#### 1.2.2 Epigenetic and transcriptional regulation

DNA methylation serves as a fundamental mechanism controlling the tissue-specific expression of CELF4. In endometrial cancer tissues, the CELF4 promoter is markedly hypermethylated, and the degree of epigenetic silencing correlates closely with tumor pathological grade; a clinical threshold of ΔCt CELF4 ≤ 8.8 has been established to distinguish benign from malignant endometrial lesions (Qi et al., 2023; Hu et al., 2026). Specific microenvironmental factors can also drive its transcription. In cultured cardiac fibroblasts, stimulation with 10 ng/ml TGF-β1 for 12 h significantly upregulates CELF4 protein expression (Zhang et al., 2025). In addition, chronic social defeat stress (CSDS) markedly reduces CELF4 mRNA and protein levels in the prefrontal cortex (PFC) of susceptible mice (Shen et al., 2022). Within post-transcriptional autoregulatory networks, CELF4 can bind its own 3′UTR, suggesting that it may fine-tune its own abundance through a feedback loop, although whether this self-binding directly suppresses its own translation remains to be validated (Salamon et al., 2023).

### 1.3 Physiological functions

CELF4 functions primarily as a translational repressor that maintains cellular structural development and excitatory homeostasis. Its physiological roles are markedly tissue-specific: the majority center on neural development and homeostasis, whereas in certain non-neural tissues it contributes to baseline quiescence.

#### 1.3.1 CNS development and synaptic regulation

CELF4 is an important translational regulator of synaptogenesis during embryonic neocortical development, and the scope of its regulated transcriptome is extensive. RNA immunoprecipitation sequencing (RIP-seq) analyses of human fetal neocortex at early, mid, and late gestational stages show that CELF4 specifically binds 227, 1,536, and 763 mRNA targets, respectively; a large proportion of these targets encode presynaptic and postsynaptic proteins according to SynGO annotation (Salamon et al., 2023). Cross-species analyses confirm that 102–220 evolutionarily conserved targets are shared between human and mouse developing neocortex, and these targets are highly enriched in pathways related to “synapse assembly,” “neurotransmitter secretion,” and “synaptic vesicle localization” (Salamon et al., 2023).

Immunoelectron microscopy reveals that CELF4 protein localizes to early synaptic contacts in the subplate (SP) zone of mouse E15.5/E17.5 and human 19 post-conceptional week (PCW) neocortex. During mammalian neocortical development, CELF4 is highly expressed in the SP zone and deep-layer excitatory neurons (L5/6), where it directly binds the 3′UTRs of mRNAs encoding synaptic proteins such as SV2A, SYP, GABRA3, and SYNPR, thereby limiting their excessive synthesis and ensuring proper synapse formation and structural integrity (Salamon et al., 2023). In the PFC, CELF4 is also physiologically required for the maintenance of dendritic spine density and synaptic structural integrity; its reduction leads to decreased spine density (Shen et al., 2022).

#### 1.3.2 Homeostatic restriction of intrinsic neuronal excitability

CELF4 serves as an endogenous “brake” that restricts neuronal hyperexcitability in both the CNS and PNS. In the CNS, wild-type CELF4 co-sediments with polysomes in the neuropil and exerts translational suppression, thereby limiting local translation of excitability-related proteins such as the voltage-gated sodium channel Nav1.6, maintaining the action-potential firing threshold, and preventing abnormal increases in persistent sodium current (Wagnon et al., 2012; Sun et al., 2013).

#### 1.3.3 Maintenance of peripheral sensory nervous system homeostasis

In the DRG, CELF4 is enriched in small-to medium-diameter sensory neurons, with particularly high expression in peptidergic (CGRP+/TRPV1+) nociceptor subpopulations and low expression in some non-peptidergic (IB4-binding) neurons. Under physiological conditions, CELF4 restricts the intrinsic excitability of nociceptors and sets the basal threshold for mechanical and thermal stimuli (Grlickova-Duzevik et al., 2023). Whole-cell patch-clamp recordings show that TRPV1+ DRG neurons from wild-type mice require relatively strong current stimulation to elicit action potentials, whereas CELF4 deficiency significantly lowers the rheobase and increases firing frequency, resulting in elevated intrinsic excitability (Mueth et al., 2025).

#### 1.3.4 Baseline homeostasis in cardiac fibroblasts

Beyond the nervous system, CELF4 is required for maintaining the quiescent state of specific peripheral cells. In cardiac fibroblasts, CELF4 binds the 3′UTR of *FMO2* mRNA to suppress its translation, thereby keeping TGF-β1/Smad2/3 signaling at a low baseline level. This translational repression mechanism is important for maintaining that cardiac fibroblasts remain in a quiescent state in healthy tissue and for preventing pathological remodeling. Notably, baseline CELF4 expression in the heart is extremely low, and the protein does not participate in the maintenance of routine cardiac function (Zhang et al., 2025; Ni et al., 2022) (Table 1).

**TABLE 1:** Tissue-specific expression and core physiological functions of CELF4.

Tissue/ Cell type	Subcellular localization	Key target mRNAs	Upstream regulators	Main physiological functions	References
Central nervous system (neurons)	Cytoplasm; neuropil; co-sedimentation with polysomes	SV2A, SYP, GABRA3, SYNPR, Scn8a (Nav1.6)	CSDS (↓) (Shen et al., 2022); Autoregulation (putative) (Salamon et al., 2023)	Local translational repression; prenatal synaptogenesis; maintenance of E/I balance and dendritic spine density	Wagnon et al. (2012), Salamon et al. (2023), Shen et al. (2022), Sun et al. (2013)
Peripheral nervous system (DRG nociceptors)	Cytoplasm (small- to medium-diameter neurons)	Scn8a (Nav1.6), Nav1.8, pronociceptive proteins	Sciatic nerve injury (↓) (Grlickova-Duzevik et al., 2023)	Restriction of intrinsic nociceptor excitability; maintenance of mechanical/thermal thresholds	Grlickova-Duzevik et al. (2023), Mueth et al. (2025)
Cardiovascular system (cardiac fibroblasts)	Cytoplasm	FMO2	TGF-β1 (↑) (Zhang et al., 2025)	Baseline repression of FMO2 translation; maintenance of fibroblast quiescence; restriction of basal TGF-β/Smad activity	Zhang et al. (2025)
Brain-gut axis (inferred)	Not determined in peripheral gut tissues	Synaptic/neuronal development genes (network inference)	Pleiotropic SNPs (genetic association)	Hub gene in brain-gut co-expression networks; potential regulator of cross-system comorbidity	Ding et al. (2025)

## 2 Roles and molecular mechanisms of CELF4 in diseases

**FIGURE 2:**
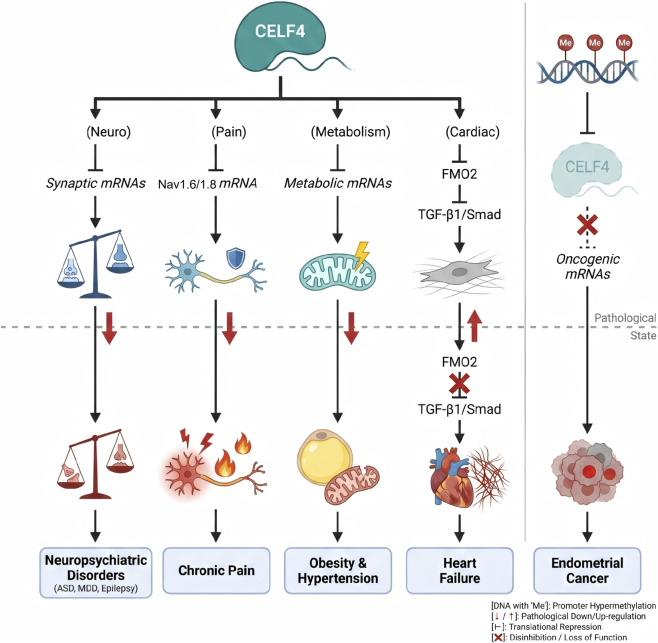
Pathophysiological landscape of CELF4-mediated regulatory networks. Physiological Homeostasis (Upper Panel): Under normal conditions, CELF4 acts as an RNA-binding protein to repress the translation of specific mRNAs, maintaining neural, sensory, metabolic, and cardiac homeostasis. Pathological Dysregulation (Lower Panel): Abnormal expression of CELF4, including haploinsufficiency (↓) or upregulation (↑), disrupts these balanced networks, driving neuropsychiatric disorders, chronic pain, obesity, and heart failure. Epigenetic Silencing (Right Panel): In endometrial tissue, promoter hypermethylation (Me) silences CELF4, lifting its translational suppression (-- ⊢ ✖) on oncogenic mRNAs and promoting tumor progression.

CELF4 exhibits diametrically opposed regulatory patterns across different disease microenvironments and models: in neuropsychiatric disorders, chronic pain, and certain tumors, it predominantly shows loss of function through downregulation or epigenetic silencing; in cardiac stress remodeling, it shows aberrant activation through pathological upregulation (Figure 2).

The mechanistic sections below vary substantially in experimental support. We categorize evidence into three levels: Level I, direct validation via conditional knockout, electrophysiology, reporter assays, or clinical biomarker cohorts; Level II, indirect inference from genetic association, behavioral rescue, or single-model observations; and Level III, bioinformatic prediction or single-pedigree observation awaiting functional confirmation. The evidence grading for each disease discussed is summarized in Table 2.

**TABLE 2:** Evidence grading and translational maturity for CELF4-Associated diseases.

Disease/ Syndrome	Core claim	Evidence level	Key experimental model	Functional validation	Translational stage	Critical bottleneck	Required validation
ASD/ NDDs	CELF4 haploinsufficiency → synaptic dysgenesis	I-II	Human variants (n = 15); Celf4 cKO (Emx1-Cre)	Polysome profiling; snRNA-seq; synaptoneurosome blot	Preclinical	Cell-type-specific KO missing; human post-mortem validation lacking	Direct measurement of CELF4 protein/RNA in post-mortem ASD patient PFC; developmental-stage-specific cKO to dissociate embryonic vs. adult functions
Epilepsy	CELF4 loss → Scn8a derepression → hyperexcitability	I-II	Celf4 global KO; human heterozygous variants	Patch-clamp; polysome profiling; immunofluorescence	Preclinical	Population case-control studies pending	Large-scale case-control cohort; conditional KO in excitatory neurons (L5/6) to confirm cell-autonomous seizure mechanism
MDD	CELF4 downregulation → dendritic spine loss	II	CSDS mouse; AAV-shCelf4 in PFC	Golgi staining; behavioral rescue (sucrose preference, TST)	Hypothetical	Single-laboratory evidence; upstream GR regulation speculative	Replication in CUMS model; post-mortem human PFC tissue; ChIP-seq or reporter assay for GR-CELF4 promoter interaction
Chronic pain	CELF4 loss → nociceptor hyperexcitability	I	Avil-CreERT2 DRG cKO	Patch-clamp; behavioral testing (mechanical/thermal hyperalgesia)	Preclinical	Subtype-specific KO unavailable	Trpv1-Cre or IB4-lineage subtype-specific KO; rescue of established chronic pain by CELF4 overexpression
Cardiac fibrosis/ HF	CELF4 upregulation → FMO2 suppression → TGF-β1/Smad2/3 activation	II	Global Celf4 KO + TAC	Dual-luciferase reporter; Masson staining; CF migration/proliferation assays	Proof-of-concept	Global KO confounds	Cardiac fibroblast-specific conditional KO; human failing heart tissue validation; competitive RBP binding assays
EC/ AH	CELF4 promoter hypermethylation → transcriptional silencing	I (epigenetic); III (mechanism)	Clinical cohort (n = 276)	MSP; qRT-PCR; ΔCt threshold validation	Clinical validation	Downstream targets unvalidated	CELF4 overexpression/knockdown in EC cell lines (proliferation, migration, clonogenic assays); CLIP-seq or RIP-seq in endometrial epithelial cells
IBS-psychiatric	CELF4 as pleiotropic hub in brain-gut axis	III	MTAG; WGCNA; PPI network inference	In silico only	Hypothetical	No intestine-specific KO	Intestine-specific knockout model; CELF4 expression mapping in human gut tissue by ISH/IHC
Obesity-hypertension	CELF4 regulatory-domain disruption → central energy dysregulation	III	Balanced translocation pedigree (n = 4 carriers)	qPCR; Hi-C; allele-specific expression	Case report	Single pedigree	Hypothalamic CELF4 expression assessment in carriers; additional pedigree or large-scale GWAS replication

### 2.1 Translational derepression and synaptic homeostatic imbalance

Loss of CELF4 function relieves translational repression of its target mRNAs. The consequent overexpression of synaptic proteins or excitatory ion channels directly disrupts excitatory-inhibitory (E/I) balance in neural circuits, ultimately producing a broad spectrum of neural phenotypes ranging from early developmental delay to adult-onset emotional and pain abnormalities.

#### 2.1.1 Neurodevelopmental disorders and autism spectrum disorder (ASD)

Neurodevelopmental disorders are a heterogeneous group of neuropsychiatric conditions with early developmental onset, arising from abnormal brain development. They include intellectual disability, ASD, and attention-deficit/hyperactivity disorder, and are characterized by impaired cognitive, social, sensory, and motor function, often with comorbid epilepsy; etiology involves both genetic and environmental factors (Ni et al., 2022; Ćuk et al., 2024; Compañ-Gabucio et al., 2024; Hori et al., 2021; de Lima et al., 2023). ASD is typified by deficits in social communication and repetitive, stereotyped behaviors; abnormal mid-gestational neocortical synaptic development and dysregulated translation of RNA-binding proteins constitute important molecular underpinnings (Griff et al., 2023; Casseus, 2022; Zhao et al., 2022; Scorrano et al., 2024; Seigfried and Britsch, 2024).

At the genetic level, CELF4 has been identified as an ASD risk gene by multiple large-scale exome sequencing studies (Bruel et al., 2025). Single-nucleus RNA sequencing (snRNA-seq) reveals that CELF4-high neuronal clusters in the human fetal frontal neocortex overlap extensively with known ASD risk-gene-enriched subplate (SP) clusters (Salamon et al., 2023). Clinical cohort data further support the pathogenicity of altered CELF4 dosage: patients with 18q12.2 microdeletion syndrome that includes *CELF4* display characteristic autistic traits (Barone et al., 2017). In an independent cohort of 15 individuals harboring heterozygous CELF4 variants (nonsense, missense, splice-site, and frameshift), the phenotypic spectrum included global developmental delay/intellectual disability (92%), epilepsy (64%), and obesity (71%), with several cases meeting diagnostic criteria for ASD (Bruel et al., 2025).

At the molecular level, *Celf4* conditional knockout (cKO) mouse models demonstrate a classic state of translational derepression in neocortical synaptic mRNAs. Polysome profiling shows that 145 mRNA isoforms are aberrantly over-translated after knockout; these derepressed targets are significantly enriched in pathways related to “synaptic vesicle priming regulation” and “presynaptic active zone components” (Salamon et al., 2023). Immunofluorescence and synaptoneurosome blot analyses confirm that SV2A and SYP proteins are markedly increased in the SP zone, whereas total cortical lysates show no difference, indicating that CELF4 primarily regulates local translation rather than global transcription (Salamon et al., 2023). This phenotype exhibits sexual dimorphism: male mice show increases in both GABAergic (VGAT+/Gphn+) and glutamatergic (vGlut2+/PSD95+) synapses in the SP zone, whereas females show reduced GABAergic synapses and immature glutamatergic synapses, offering a possible molecular explanation for the male predominance in ASD (Salamon et al., 2023).

Notably, the polysome profiling data (Salamon et al., 2023) and the human variant data (Bruel et al., 2025) converge on synaptic dysregulation, yet the human cohort lacks post-mortem brain tissue validation, leaving a gap between genetic association and molecular mechanism.

Nevertheless, several challenges remain. Clinical variant evidence derives largely from single-center, small cohorts or case reports and lacks large-scale population validation (Level I-II). Moreover, the Emx1-Cre driver used in existing cKO mice initiates knockout at embryonic day 9.5 (E9.5), making it difficult to dissociate the independent contributions of “developmental synapse formation defects” from “adult synaptic maintenance dysfunction.” The peak CELF4 expression window in human fetal neocortex (∼19 PCW, mid-gestation) (Salamon et al., 2023) precedes the typical clinical ASD diagnosis window (>24 months) by approximately 2 years, creating a temporal mismatch that limits postnatal intervention opportunities. The drivers behind this sexual dimorphism---for example, whether there is crosstalk with sex-hormone receptor pathways---also remain to be elucidated.

#### 2.1.2 Major depressive disorder (MDD)

Major depressive disorder is a common and disabling mood disorder affecting approximately 264–350 million people worldwide (Meneses-San et al., 2023; Mahmud et al., 2023). Chronic stress-induced reduction in dendritic spine number in the PFC is an important cellular basis for dysfunctional neural circuits in depression (Shinohara and Furuyashiki, 2025; Algaidi, 2025; Bulek and BaDour, 2026; Merz et al., 2023).

Acquired downregulation of CELF4 is closely associated with depressive-like degenerative changes. In the CSDS mouse model, susceptible individuals show significantly lower CELF4 protein and mRNA levels in the PFC than resilient individuals, and this trend parallels the classic depression biomarker BDNF (Shen et al., 2022). This downregulation is closely associated with synaptic structural damage: Golgi staining reveals markedly reduced dendritic spine density in the PFC of CSDS-susceptible mice. AAV-mediated sh*Celf4* knockdown in the PFC recapitulates the spine-loss phenotype and induces anhedonia (reduced sucrose preference), behavioral despair (increased immobility in the tail-suspension test), and anxiety-like behavior (decreased time in the open-field center) (Shen et al., 2022). Given that CELF4 can regulate the translation of synaptic-function-related mRNAs, its reduced abundance in the PFC may disrupt the equilibrium of local translation of key synaptic proteins, thereby triggering dendritic spine degeneration.

The parallel between CELF4 downregulation and BDNF reduction (Shen et al., 2022) suggests a convergent stress-response pathway, but whether CELF4 acts upstream or downstream of BDNF, or whether they represent independent branches, remains unresolved.

Currently, evidence linking CELF4 to depression derives from a single laboratory’s mouse model and urgently requires cross-validation in multiple depression models (e.g., chronic unpredictable mild stress, CUMS) and in post-mortem human brain tissue (Level II). More critically, the specific downstream targets of CELF4 in the PFC and the upstream regulatory pathways that control its expression under stress remain undefined. Given the association between glucocorticoid receptor (GR) activation and PFC synaptic atrophy, GR-mediated transcriptional suppression of CELF4 is a testable hypothesis; however, no ChIP-seq, reporter assay, or GR-binding motif analysis currently supports direct GR-CELF4 promoter interaction. In addition, cell-type-specific functions of CELF4 in distinct neuronal subtypes remain unresolved.

#### 2.1.3 Epilepsy

Epilepsy is a common neurological disorder characterized by unprovoked recurrent seizures and electroencephalographic abnormalities, affecting all age groups (Petralla et al., 2026; Frauscher et al., 2024; Aldosary et al., 2021; P et al., 2024). Its core pathological basis is dysfunctional brain network activity, in which ion-channel abnormalities mediate neuronal hyperexcitability, leading to synaptic E/I imbalance and aberrant neuronal synchronization (Chen et al., 2024; Kuang et al., 2023; Liu X. et al., 2024; Liu C. et al., 2024).

In both model animals and human patients, loss of CELF4 function has been established as a potent trigger for epileptic seizures. Global *Celf4* knockout mice exhibit complex seizures, including convulsive and non-convulsive episodes, attributable to markedly increased neuronal excitability (Wagnon et al., 2011; Yang et al., 2007). Electrophysiological recordings pinpoint the cellular mechanism: in layer V pyramidal neurons lacking *Celf4*, the action-potential threshold is lowered, gain is increased, persistent sodium current is enhanced, and voltage dependence shifts in the hyperpolarizing direction. The molecular basis identified is that CELF4 normally binds *Scn8a* (encoding Nav1.6) mRNA and restricts its translation; *Celf4* deletion causes abnormal accumulation of Nav1.6 protein, directly elevating excitability (Sun et al., 2024). This mechanism is consistent with clinical observations: in a cohort of 15 patients with heterozygous CELF4 variants, 64% (9/15) exhibited epilepsy, with onset ages ranging from 8 months to 8 years and seizure types spanning febrile seizures to epileptic encephalopathy (Bruel et al., 2025); similar susceptibility is observed in patients with 18q12.2 microdeletions (Barone et al., 2017; Halgren et al., 2012).

Furthermore, the true genetic contribution of CELF4 variants to epilepsy in patient populations still requires assessment through large-scale case-control studies (Level I-II). At the mechanistic level, whether the epileptic phenotype in *Celf4* knockout mice is fully mediated by Nav1.6 overabundance or involves cooperative effects with other voltage-gated ion channels (e.g., Nav1.2, Nav1.8) and synaptic receptors remains to be dissected. More importantly, the dynamic trajectory of CELF4 expression across different stages of epileptogenesis (pre-ictal, acute, and chronic remodeling) has not been fully mapped, limiting our understanding of its temporal role in seizure genesis.

#### 2.1.4 Chronic pain

Chronic pain affects approximately 20% of adults worldwide; nociceptor neuroplasticity and post-transcriptional regulation of pain-related mRNAs by RNA-binding proteins are central to peripheral sensitization (Li et al., 2021; Li et al., 2022; Puts et al., 2024; Kunder et al., 2022; Xiong et al., 2024).

In the peripheral sensory system, CELF4 serves as an endogenous excitability-limiting factor that maintains basal thresholds for mechanical and thermal stimuli. Histological sequencing and immunofluorescence quantification show that CELF4 is enriched in small-to medium-diameter DRG neurons, with particularly high expression in peptidergic (CGRP+/TRPV1+) nociceptor subpopulations (58% of small-diameter and 38% of medium-diameter neurons); retrograde tracing further confirms that 76% of TRPV1+ muscle afferent fibers co-express CELF4 (Grlickova-Duzevik et al., 2023). DRG-specific knockout driven by Avil-CreERT2 demonstrates that CELF4 deficiency induces marked hyperexcitability in TRPV1+ DRG neurons: action-potential firing frequency increases and the rheobase decreases by approximately twofold (Grlickova-Duzevik et al., 2023). Behavioral testing shows that knockout mice exhibit sustained and significant mechanical and thermal hyperalgesia; notably, CELF4 loss places the nociceptive pathway in a “pre-sensitized” state---microdoses of nerve growth factor (NGF, 5 ng) or capsaicin (0.01%), equivalent to one-tenth the conventional dose, induce significant hyperalgesia in knockout mice that is absent in wild-type controls (Mueth et al., 2025). In the early phase (days 1–7) of neuropathic pain (sciatic nerve injury), CELF4 expression in the DRG is significantly downregulated, which may relieve translational repression of ion channels (e.g., Nav1.6, Nav1.8) and pronociceptive proteins (Grlickova-Duzevik et al., 2023).

The upstream regulatory pathways that drive CELF4 downregulation in pain models have not been clarified. Because existing functional studies have used Avil-Cre-mediated whole-DRG knockout, the respective contributions of CELF4 in peptidergic versus non-peptidergic neurons cannot be distinguished (Level I). Future studies should employ Trpv1-Cre or IB4-lineage tracing to dissociate peptidergic from non-peptidergic contributions and map subtype-specific translational targets. Moreover, whether enhancing CELF4 function can substantially reverse established chronic pain remains to be tested.

Although the above neurological diseases differ in phenotype, they share a core pathway: CELF4 loss → translational derepression of synaptic proteins/ion channels → E/I imbalance. However, ASD primarily affects developmental synaptogenesis, MDD primarily affects adult synaptic homeostasis, and epilepsy and chronic pain involve central and peripheral excitability thresholds, respectively. This spatiotemporal specificity indicates that the pathological effects of CELF4 are highly dependent on developmental stage and cell type and cannot be fully captured by a simplistic loss-of-function model.

### 2.2 Cytokine signaling reprogramming and tissue fibrosis

In contrast to its downregulation in the nervous system, CELF4 exhibits non-physiological transcriptional activation in certain non-neural tissues under pathological stimulation. By restructuring matrix-related signaling pathways, it becomes a important contributor of tissue fibrosis.

#### 2.2.1 Heart failure and cardiac fibrosis

Cardiac remodeling in heart failure is characterized by pathological myocardial hypertrophy, cardiac fibroblast (CF) activation, and extracellular matrix deposition; excessive activation of the TGF-β1/Smad signaling pathway plays a central role (Li et al., 2023; Frangogiannis, 2024; Humeres et al., 2024; Chakrabarti et al., 2023; Deng et al., 2025).

In a mouse cardiac remodeling model induced by pressure overload (transverse aortic constriction, TAC), CELF4 protein---which is normally expressed at extremely low levels---begins to rise by postoperative day 3 and peaks at day 10 (Zhang et al., 2025). Cellular analyses confirm that this upregulation is specifically enriched in CFs; *in vitro* experiments reveal the upstream trigger: stimulation of CFs with 10 ng/ml TGF-β1 for 12 h significantly induces CELF4. Functionally, global *Celf4* knockout mice subjected to TAC show significant improvements in cardiac function and remodeling indices after 4 weeks: left ventricular ejection fraction (LVEF) and fractional shortening (FS) recover, left ventricular internal dimensions (LVIDd, LVIDs) decrease, and Masson staining shows a ∼26% reduction in fibrotic area (from 8.43% to 6.20%, P < 0.01) (Zhang et al., 2025). In *ex vivo* experiments, CELF4 deficiency markedly suppresses TGF-β1-induced CF migration and proliferation and reduces α-SMA and collagen I expression. Although these data establish a cardioprotective correlation, the use of global knockout precludes attribution of the phenotype to cell-autonomous CF function versus systemic compensatory effects from immune or vascular cells.

Molecular studies have established the core profibrotic pathway: CELF4 directly binds the 3′UTR of *FMO2* mRNA to suppress its translation. Dual-luciferase assays show that this inhibitory effect specifically depends on the Mut2-FMO2 region within the 3′UTR. FMO2 is an endogenous negative regulator of the TGF-β/Smad pathway that suppresses Smad2/3 phosphorylation by promoting SMURF2 expression (Ni et al., 2022); by inhibiting FMO2 translation, CELF4 relieves this negative feedback brake, allowing sustained activation of TGF-β1/Smad2/3 signaling (Zhang et al., 2025).

Because all existing evidence derives from global knockout mice, the development of cardiac fibroblast-specific conditional knockout models is urgently needed to exclude systemic compensatory effects and establish definitive cell-autonomous profibrotic function (Level II). In addition, whether FMO2 has other critical downstream nodes besides SMURF2, and whether the CELF4-FMO2 interaction is subject to competitive regulation by other RNA-binding proteins, remain to be clarified. The expression profile of this novel mechanism in human heart failure samples and its correlation with clinical prognosis also require validation.

### 2.3 Epigenetic silencing and tumorigenesis

In the oncological dimension, the core pathological mechanism of CELF4 involves aberrant hypermethylation of CpG islands in the gene promoter region. This epigenetic modification leads to transcriptional silencing, loss of translational repression of potential downstream oncogenic target mRNAs, and consequently drives malignant transformation of epithelial cells.

#### 2.3.1 Endometrial cancer (EC) and atypical hyperplasia (AH)

Endometrial cancer is the most common gynecological malignancy worldwide, and endometrial atypical hyperplasia (AH) represents a critical precancerous stage in its progression (Prip et al., 2022; Wu et al., 2021; Adjei et al., 2024; Chou et al., 2024). During endometrial malignant transformation, CELF4 displays a highly consistent pattern of epigenetic silencing. Consistent with the epigenomic landscape of EC, promoter hypermethylation of CELF4 has been repeatedly identified in clinical cohorts, with significantly lower ΔCt values in malignant versus benign tissues (Qi et al., 2023; Hu et al., 2026). This epigenetic landscape has been validated in independent clinical samples: EC and AH tissues show significantly lower CELF4 methylation levels (expressed as ΔCt values) than benign endometrial tissues, and the degree of methylation increases in a graded manner with advancing tumor pathological grade (Qi et al., 2023; Hu et al., 2026).

While CELF4 promoter hypermethylation is robustly validated in EC (Level I), its downstream translational targets in endometrial epithelial cells remain hypothetical. Unlike the neocortex, where RIP-seq has identified SV2A, SYP, and *Scn8a* as direct targets (Salamon et al., 2023), no CLIP-seq or RIP-seq has been performed in endometrial cell lines. Consequently, the tumor-suppressor function of CELF4 in EC is inferred from its canonical neural mechanism, not empirically established.

Although epigenetic silencing of CELF4 is well established, its specific downstream effector molecules as a putative tumor suppressor remain to be identified (Level I for methylation; Level III for tumor-suppressor mechanism). Based on the protein’s classic mode of action in the nervous system---binding 3′UTRs to suppress mRNA translation---it is reasonable to speculate that in physiological endometrial epithelial cells, CELF4 may similarly repress the translation of certain pro-proliferative, anti-apoptotic, or pro-inflammatory mRNAs; aberrant methylation-induced silencing would then cause derepression of these targets, initiating a malignant transformation cascade. However, this post-transcriptional remodeling hypothesis currently lacks direct *in vivo* validation from RIP-seq or CLIP-seq. Accordingly, although the direct targets of CELF4 in EC remain experimentally unverified, its recognition of UG-rich sequences suggests that it may regulate the translation of cell-cycle or apoptosis-related genes; this inference awaits testing in endometrial epithelial cell models.

In addition, whether fluctuations in CELF4 methylation status are intrinsically linked to classic EC molecular subtypes (e.g., POLE-mutant, MSI, copy-number low, and copy-number high) is unclear. At the clinical-translational level, whether intervention with DNA demethylating agents (e.g., decitabine) can restore endogenous CELF4 expression and thereby suppress tumor progression also remains to be investigated.

### 2.4 Pleiotropic genetic loci and systemic comorbidities

Unlike the marked expression changes observed in single-system or single-tissue diseases, CELF4 exhibits unique genetic characteristics in complex cross-system comorbidities. It typically does not elicit a single phenotype directly but instead acts as a hub gene or pleiotropic regulatory locus within shared genetic networks, connecting disparate pathophysiological processes (Table 3).

**TABLE 3:** Expression status and pathological mechanisms of CELF4 in various diseases.

Disease/ Syndrome	CELF4 status/ Expression	Core molecular mechanism	Phenotype/ Clinical implication	References
ASD/ Neurodevelopmental disorders	Haploinsufficiency (human CELF4); downregulated (Celf4 cKO model)	Loss of translational repression of synaptic mRNAs; sexual dimorphic synaptic imbalance	Intellectual disability, epilepsy, ASD, obesity (human); social communication deficits (model)	Bruel et al. (2025), Salamon et al. (2023), Barone et al. (2017), Halgren et al. (2012)
Epilepsy	Haploinsufficiency/ loss of function	Translational derepression of Scn8a (Nav1.6); persistent Na + current enhancement	Complex seizures, neuronal hyperexcitability; onset 8 months-8 years	Bruel et al. (2025), Sun et al. (2013), Wagnon et al. (2011), Yang et al. (2007), Halgren et al. (2012)
Major depressive disorder (MDD)	Downregulated (PFC)	Reduced dendritic spine density; impaired local translation of synaptic proteins	Anhedonia, behavioral despair, anxiety-like behavior	Shen et al. (2022)
Chronic pain	Downregulated (DRG)	Disinhibition of ion channel/pronociceptive protein translation in TRPV1+ nociceptors	Mechanical/thermal hyperalgesia; pre-sensitized nociceptive state	Grlickova-Duzevik et al. (2023), Mueth et al. (2025)
Cardiac fibrosis/ Heart failure	Upregulated (cardiac fibroblasts)	FMO2 translational suppression → sustained TGF-β1/Smad2/3 activation	Cardiac fibroblast activation, proliferation, collagen deposition	Zhang et al. (2025)
Endometrial cancer (EC)/ Atypical hyperplasia (AH)	Silenced (promoter hypermethylation)	Epigenetic silencing; putative loss of tumor suppressor function	Malignant transformation; diagnostic biomarker (ΔCt ≤ 8.8; sensitivity 84.9%)	Qi et al. (2023), Hu et al. (2026)
Obesity-hypertension comorbidity	Regulatory domain disruption (balanced translocation t (11; 18))	Altered long-range cis-regulatory architecture; central energy dysregulation	Early-onset obesity, hypertension (age-dependent penetrance)	Yang et al. (2007), Fjorder et al. (2019)
IBS-psychiatric comorbidity	Pleiotropic locus variation (genetic association)	Disrupted brain-gut shared transcriptional network (WGCNA/PPI inference)	IBS with depression/anxiety comorbidity	Ding et al. (2025)

#### 2.4.1 Comorbid mechanisms of irritable bowel syndrome (IBS) and psychiatric disorders

IBS and psychiatric disorders (e.g., depression and anxiety) are highly comorbid in clinical epidemiology and share substantial genetic underpinnings, yet the core molecular pathways driving this gut-brain axis comorbidity have long remained unclear (Li et al., 2026; Yang et al., 2026; Pop et al., 2025; Zhu et al., 2024). Through multi-trait genome-wide association analysis (MTAG), Ding and colleagues identified 60 novel pleiotropic loci for IBS in a large joint analysis of IBS with depression (DEP), major depressive disorder (MDD), and neuroticism (NE); signals near the CELF4 locus showed high cross-phenotypic stability (Ding et al., 2025). Gene enrichment analyses indicate that genes near these pleiotropic loci are significantly enriched in neuronal development and synaptic function pathways. Weighted gene co-expression network analysis (WGCNA) integrating human brain and digestive system transcriptomes further revealed multiple co-expression modules that are highly conserved between the amygdala and the esophageal muscularis or sigmoid colon (Zsummary > 20) (Ding et al., 2025). Within these brain-gut shared modules, CELF4 was identified as a putative hub gene; this status requires functional validation in intestine-specific knockout models. Protein-protein interaction (PPI) network analyses confirm that CELF4 serves as a key physical node bridging these cross-system shared gene networks (Ding et al., 2025).

It must be noted that current evidence for CELF4-mediated IBS-psychiatric comorbidity relies entirely on genetic association and bioinformatic co-expression inference and lacks *in vivo* functional validation (Level III). The specific cell-type distribution of CELF4 in intestinal tissue has not been systematically mapped, and how these pleiotropic SNPs affect CELF4 abundance or splicing variants remains to be causally tested using intestine-specific knockout animal models.

#### 2.4.2 Comorbid mechanisms of obesity and hypertension

Hypertension and obesity frequently coexist; weight gain can elevate blood pressure through neurohumoral mechanisms, yet the specific molecular genetic basis driving their comorbidity remains to be clarified (Do et al., 2023; Sindhwani et al., 2026). Genomic rearrangements involving CELF4 have also been implicated in metabolic-cardiovascular comorbidity. In a rare Danish pedigree carrying a balanced translocation t (11; 18) (q22.1; q12.2), four adult carriers exhibited early-onset hypertension (onset age 35–48 years) and severe obesity (BMI 34–43 kg/m^2^); a 23-year-old younger carrier had normal indices, suggesting age-dependent penetrance, whereas relatives without this karyotype were phenotypically normal (Fjorder et al., 2019).

Fine-mapping of the molecular breakpoints revealed the genetic basis of this comorbidity. Mate-pair and Sanger sequencing showed that the chromosome 11 breakpoint lies within intron four of the hypertension-related gene *ARHGAP42*, whereas the chromosome 18 breakpoint falls precisely within the regulatory domain upstream of *CELF4* (without disrupting the CELF4 coding sequence) (Fjorder et al., 2019). Quantitative PCR and allele-specific expression analyses confirmed that *ARHGAP42* mRNA expression in peripheral blood is reduced by approximately 50% through nonsense-mediated decay. Regarding the chromosome 18 abnormality, Hi-C heatmap analyses reveal that the CELF4 locus resides within a topologically associating domain (TAD) spanning 5 Mb in the 18q12.2 region; this domain is densely populated with multiple obesity-related GWAS signals (e.g., rs4327120, rs9304204, rs7226835) (Fjorder et al., 2019). Capture-HiC confirmed active chromatin three-dimensional interactions between these SNP regions and the CELF4 promoter (Fjorder et al., 2019). Thus, the chromosomal break may disrupt the long-range cis-regulatory architecture of CELF4, potentially altering CELF4 expression in central energy-regulatory networks; however, direct evidence of CELF4 transcript or protein dysregulation in carriers’ hypothalamic tissue is lacking, and the obesity phenotype may involve additional cis-regulatory elements within the TAD.

For hypertension in this pedigree, it is currently difficult to strictly separate the respective contributions of ARHGAP42 haploinsufficiency (direct causation) from CELF4 regulatory-domain disruption-mediated obesity (secondary elevation of blood pressure) (Level III). Moreover, because CELF4 is physiologically not expressed in adult non-neural peripheral tissues, its targeted transcriptional changes cannot be directly assessed in peripheral blood samples. Because evidence currently derives solely from this single isolated pedigree, future replication in additional large-scale cohorts is required to firmly establish the causal role of CELF4 in systemic metabolic remodeling.

## 3 Clinical translation and intervention strategies

Current translational research on CELF4 is advancing along three distinct paths: as a non-invasive diagnostic and screening biomarker, as a pathogenic benchmark for rare-disease genetic counseling, and as a potential pharmacological target for systemic diseases. However, apart from tumor methylation testing, which has entered clinical validation, most targeted intervention strategies remain at the proof-of-concept stage, and clinical translation continues to face substantial barriers related to delivery systems, drugability, and safety.

### 3.1 Development of diagnostic and screening biomarkers

#### 3.1.1 Epigenetic molecular diagnosis of endometrial cancer

Among all translational directions for CELF4, methylation testing for endometrial cancer (EC) and its precancerous lesion (AH) is the most mature and clinically promising. Clinical studies have established a threshold of ΔCt CELF4 ≤ 8.8 for methylation positivity. In a prospective cohort of 276 women with indications for endometrial biopsy, the median CELF4 ΔCt in the malignant group (AH + EC) was significantly lower than in the benign group (6.89 vs. 11.8, P < 0.001). Based on this threshold, single-gene testing achieved a sensitivity of 84.9%, a specificity of 86.6%, and an AUC of 0.86 for AH/EC (Qi et al., 2023).

To further improve diagnostic performance, multi-gene panels and machine-learning algorithms have shown enhanced clinical value. Studies indicate that dual-gene testing based on CDO1 and CELF4 is an independent risk factor for EC (OR = 6.92) (Qi et al., 2023); an XGBoost diagnostic model combining CELF4 with HTR1B and TBX5 improves overall accuracy to 90% and sensitivity to 97% (Hu et al., 2026). In postmenopausal women, combining this methylation test with routine transvaginal ultrasound (endometrial thickness ≥ 5 mm) yields a specificity of 88.8%–94.9% (Qi et al., 2023). Based on these findings, a methylation detection kit integrating CDO1 and CELF4 (e.g., CISENDO®) has been approved for clinical investigation in China; wider adoption of this strategy may reduce unnecessary diagnostic curettage and other invasive procedures (Wang et al., 2022).

Nevertheless, several methodological and epidemiological bottlenecks must be resolved before large-scale clinical implementation. First, the excellent performance reported to date was achieved in highly suspected populations (abnormal bleeding or ultrasound findings); its real-world screening efficacy in asymptomatic general populations awaits validation through large-scale, multicenter, prospective cohorts. Second, standardized quality-control systems for ΔCt thresholds across different laboratories are urgently needed. In addition, whether CELF4 methylation status can guide EC molecular subtyping or predict sensitivity to specific chemotherapeutic or immunotherapeutic regimens remains unknown.

#### 3.1.2 Physical barriers to neuropsychiatric biomarkers

In stark contrast to advances in oncology, biomarker translation for CELF4 in neuropsychiatric disorders encounters inherent biological limitations. Although its abnormal expression in the CNS has been repeatedly confirmed in post-mortem brain tissue and animal models, the fact that CELF4 is virtually absent from accessible peripheral tissues such as blood, saliva, or urine after birth completely blocks the path toward direct use of its protein or transcript as a liquid biopsy marker (Grlickova-Duzevik et al., 2023; Salamon et al., 2023; Shen et al., 2022). Future development of functional imaging probes targeting key downstream synaptic proteins within the CELF4 regulatory network, or identification of relevant miRNAs in peripheral blood mediated by brain-derived exosomes, may offer alternative strategies to circumvent this peripheral non-expression barrier.

### 3.2 Development prospects and pharmacological challenges for targeted interventions

Because CELF4 exhibits heterogeneous loss of function or aberrant activation across different diseases, its pharmacological targeting must adopt highly disease-customized strategies, raising distinct safety and delivery challenges in each context.

#### 3.2.1 Chronic pain and epilepsy: Activating the endogenous molecular brake

In chronic pain and epilepsy models, loss of CELF4 leads to disruption of electrophysiological thresholds. Therefore, restoring or enhancing its translational repressor function is viewed as a potential therapy to re-establish E/I balance. Mueth and colleagues explicitly proposed that activating this “endogenous limiting factor” may represent a novel analgesic concept (Mueth et al., 2025). Animal experiments also show that abnormal phenotypes in knockout mice peak at day 7 and partially recover spontaneously by day 21, suggesting that the CELF4-mediated regulatory network is reversible and offering a therapeutic time window for pharmacological intervention (Mueth et al., 2025); the same mechanism applies to correcting refractory epilepsy caused by *Celf4* deficiency (Wagnon et al., 2011).

However, developing positive modulators of CELF4 faces significant drugability challenges. As a canonical RNA-binding protein, CELF4 lacks the classic small-molecule binding pockets characteristic of kinases and other traditional drug targets; directly enhancing its RNA-binding activity with chemical small molecules is extremely difficult. Potential strategies include screening for stabilizers that block CELF4 ubiquitination and degradation, or using antisense oligonucleotides (ASOs) to antagonize endogenous negative regulators upstream of CELF4. More pressing safety concerns remain: systemic activation of CELF4 could excessively suppress cortical synaptic plasticity, potentially inducing cognitive slowing or motor deficits. Therefore, highly organ-specific delivery technologies will be essential in the future.

#### 3.2.2 Cardiac fibrosis: Targeted inhibition via nucleic acid drugs

In contrast to the nervous system, CELF4 shows aberrant profibrotic activation in cardiac fibroblasts under pressure overload. Using siRNAs or ASOs targeting CELF4 mRNA to specifically knock down the gene in CFs can relieve FMO2 suppression and thereby block the TGF-β1/Smad2/3 pathway, offering a new perspective for reversing heart failure remodeling (Zhang et al., 2025). Given the extremely low baseline expression of CELF4 in the cardiovascular system under physiological conditions, the off-target risk and safety margin for targeted inhibition are theoretically far more favorable than in neurological diseases. Before clinical advancement, however, this strategy still lacks critical evidence. The cardioprotective efficacy reported to date derives entirely from global knockout mice, which cannot completely exclude compensatory contributions from other peripheral systems such as immune cells; because all existing evidence derives from global knockout mice, the development of cardiac fibroblast-specific conditional knockout models is urgently needed to exclude systemic compensatory effects and establish definitive cell-autonomous profibrotic function. Concurrently, developing delivery vehicles with high tropism for cardiac fibroblasts is a core prerequisite for translating this nucleic acid therapy.

#### 3.2.3 Neurodevelopmental disorders: Time-window limitations for gene replacement therapy

For 18q12.2 microdeletion syndrome or ASD caused by CELF4 haploinsufficiency, the most direct strategy is gene replacement therapy. However, the translation of such prenatal developmental disorders faces severe time-window limitations. Studies indicate that CELF4 plays an irreplaceable role in prenatal neocortical synaptic development (Salamon et al., 2023), implying that the optimal window for genetic intervention is likely restricted to the embryonic period or the very early postnatal phase, whereas most patients with neurodevelopmental disorders (NDDs) are diagnosed only after critical periods of synaptic development have largely concluded. Specifically, CELF4 expression peaks at ∼19 PCW (Salamon et al., 2023), whereas ASD diagnosis typically occurs after 24 months of age, leaving a postnatal gap of approximately 2 years during which critical periods of synaptic development may have largely concluded. Moreover, large-scale AAV intracranial injection in non-lethal neuropsychiatric diseases faces high ethical review barriers regarding long-term safety and immunogenicity. For the small subset of patients carrying nonsense or splice-site mutations, nonsense-mutation readthrough drugs (e.g., ataluren) or ASO-mediated exon skipping may represent more clinically feasible alternatives (Bruel et al., 2025).

### 3.3 Clinical genetic counseling and prenatal diagnostic alerting for rare variants

In the field of rare genetic disease, 18q12.2 microdeletions/microduplications encompassing CELF4 and single-gene pathogenic variants have been brought into the scope of clinical genetic counseling. According to the gnomAD database, CELF4 has a pLI score of 1.00 and a LOEUF score of 0.23, indicating extreme intolerance to loss-of-function (LoF) variants. Under ACMG/AMP guidelines, its LoF variants (nonsense, frameshift, and canonical splice-site disruption) carry strong pathogenic weight (Bruel et al., 2025).

Prenatal diagnosis, however, faces a challenge of being “detectable but difficult to predict.” Although amniocentesis chromosomal microarray (CMA) or whole-genome sequencing (WGS) can capture copy-number variations at 18q12.2 *in utero*, the clinical penetrance of deletions in this region is incomplete, and fetal ultrasound imaging typically reveals no obvious structural abnormalities (Barone et al., 2017). Postnatal phenotypic heterogeneity is broad, ranging from mild isolated obesity or learning disability to composite syndromes with refractory epilepsy and severe ASD (Barone et al., 2017; Halgren et al., 2012). This phenotypic variability complicates prenatal genetic counseling and may lead to over-medicalization or unnecessary pregnancy termination. Because CELF4 lacks physiological expression in adult peripheral blood, pathogenicity assessment for missense variants cannot rely on routine patient blood testing and currently depends primarily on familial co-segregation data and *in silico* prediction (Bruel et al., 2025), highlighting the need to develop neural organoids for clinical variant functional validation.

### 3.4 A translational roadmap: From biomarker detection to precision intervention

Current Section 3 has focused heavily on describing the *status quo* of individual diseases, yet several cross-cutting gaps remain unaddressed. First, no diagnostic-therapeutic closed loop exists: for patients screening positive for CELF4 methylation (ΔCt ≤ 8.8) in EC, there are currently no guidelines on whether to initiate CELF4-functional intervention or downstream FMO2-targeted therapy, nor is there a stratification algorithm (e.g., whether ΔCt < 5.0 warrants more aggressive adjunctive therapy). Second, organ specificity remains the dominant safety bottleneck. Systemic activation of CELF4 to treat neurological disorders (Section 3.2.1) could theoretically enhance cardiac fibroblast CELF4 expression, albeit from an extremely low baseline, and thereby inadvertently promote fibrosis via the FMO2-Smad2/3 axis described in Section 2.2.1. Conversely, systemic inhibition of CELF4 for cardiac fibrosis must rigorously exclude off-target neural suppression. These bidirectional risks mandate cell-subtype-specific delivery.

Delivery technology comparison. For CNS indications, intracranial AAV with neuron-specific promoters (Syn1/hSyn) is the most mature platform, but its invasiveness raises ethical barriers for non-lethal NDDs. Focused ultrasound (FUS) combined with microbubbles can transiently and reversibly open the blood-brain barrier to facilitate intravenous AAV delivery to deep structures (e.g., PFC), reducing surgical risk, yet it does not resolve neuronal-subtype specificity. For DRG-targeted pain therapy, intrathecal ASO injection achieves regional restriction but requires repeated dosing. Engineered exosomes surface-modified with targeting ligands (e.g., RVG peptide for neural tissue, cardiac-homing peptide for CFs) offer reduced immunogenicity and improved cell tropism, but manufacturing scalability and loading efficiency remain bottlenecks. Table 4 summarizes the optimal intervention strategy, preferred delivery platform, core safety endpoints, and preclinical validation models for each disease category.

**TABLE 4:** Translational roadmap for CELF4-Targeted strategies.

Disease category	Optimal intervention strategy	Preferred delivery platform	Core safety endpoints	Preclinical validation models	Key bottlenecks
ASD/ NDDs	Gene replacement (AAV-hSyn-CELF4) or nonsense readthrough (ataluren)	Intracranial AAV with neuron-specific promoter (Syn1/hSyn); FUS + microbubbles for transient BBB enhancement to reduce invasiveness	Cognitive/motor slowing; systemic off-target to cardiac fibroblasts; immune response	Celf4 cKO; iPSC-derived neural organoids	∼2-year temporal gap (19 PCW vs > 24 months diagnosis); ethical barriers for intracranial AAV in non-lethal NDDs
Epilepsy/ Chronic pain	Positive allosteric modulator of CELF4-RNA binding, or ASO against upstream suppressor	Intrathecal ASO for DRG/PFC regional delivery; engineered exosomes for nociceptor subtype targeting	Nociceptor hypoexcitability; CNS depression; unintended cardiac fibroblast activation	Avil-CreERT2 DRG cKO; PFC shRNA	Druggability of RBP interface; reversibility window (day 7 peak, day 21 partial recovery); peptidergic vs non-peptidergic specificity
Cardiac fibrosis/ HF	siRNA/ ASO targeting CELF4 in CFs	CF-tropic AAV or engineered exosomes (surface-modified for cardiac fibroblast homing)	Baseline cardiac function; off-target neural suppression (if systemic); immune response	CF-specific conditional KO (currently unavailable; global KO used as surrogate)	Global KO confounds prevent cell-autonomous attribution; CF-specific delivery vehicles remain immature
EC/ AH	DNA demethylating agents (e.g., decitabine) to restore CELF4 expression	Systemic (oral/ IV)	Myelosuppression; global hypomethylation off-target effects	EC cell lines (Ishikawa, HEC-1A) + xenografts	No validated downstream targets in EC; no biomarker-guided treatment stratification (e.g., whether ΔCt <5.0 warrants FMO2-targeted adjunctive therapy)

## 4 Summary and perspectives

CELF4 is a neural-enriched RNA-binding protein that functions as a molecular node in multisystem pathophysiology. Under physiological conditions, it acts primarily as a translational repressor in the nervous system to maintain E/I balance and sensory homeostasis, whereas it is expressed at low levels in non-neural tissues. Under pathological stress, CELF4 exhibits bidirectional regulation: in neurodevelopmental disorders, depression, chronic pain, and endometrial cancer, it shows loss of function through downregulation, haploinsufficiency, or promoter hypermethylation; in cardiac fibrosis, it is aberrantly induced by TGF-β1 and other factors, suppresses FMO2, and shows aberrant activation. Additionally, CELF4 acts as a pleiotropic genetic regulatory locus involved in gut-brain axis and metabolic-cardiovascular comorbidity networks. Currently, endometrial cancer screening based on CELF4 hypermethylation offers the strongest clinical translational prospect; its haploinsufficiency has been incorporated into the clinical genetic diagnosis of rare diseases; and pharmacological interventions and gene replacement therapies targeting CELF4 remain at the preclinical proof-of-concept stage.

Model and population limitations. Although a preliminary multisystem molecular atlas of CELF4 has been established, several basic and clinical bottlenecks persist. At the level of basic regulatory mechanisms, the complete RNA target spectra of CELF4 in cardiac fibroblasts, endometrial epithelial cells, and specific DRG subpopulations remain unmapped; future studies should apply CLIP-seq and RIP-seq broadly to fill this gap. Concurrently, the upstream trigger networks that cause CELF4 downregulation after chronic stress or upregulation during fibrosis---such as receptor-mediated signaling or epigenetic regulation---await elucidation. Moreover, whether the sexual dimorphism in CELF4-mediated translational regulation observed in mice is directly controlled by sex-hormone pathways, and what contribution this makes to the higher male prevalence of human autism, are research directions worthy of in-depth investigation.

Regarding model construction and population genetic evidence, existing functional validations rely predominantly on global knockout or local brain-region knockdown, making it difficult to dissociate systemic compensatory effects. The field should accelerate the construction of conditional knockout and knock-in models targeting nociceptors, cardiac fibroblasts, and specific neuronal subpopulations to precisely dissect cell-autonomous functions. At the population level, large-scale diversified cohorts beyond European ancestry are urgently needed to validate genetic associations between CELF4 variants and cross-system comorbidities and to correct for racial bias; we also call for the establishment of an international multicenter phenotypic registry for 18q12.2 microdeletion syndrome to clarify its true penetrance and genotype-phenotype mapping.

Intervention and translation priorities. At the level of intervention innovation and translation, CELF4 methylation diagnostic kits must complete prospective real-world efficacy assessments in asymptomatic populations and establish standardized quality-control systems. For drug-target development, future priorities should include high-throughput screening and surface plasmon resonance (SPR) technologies to develop small-molecule allosteric modulators that specifically block the CELF4-target RNA binding interface; delivery systems also require optimization, such as intrathecal ASO injection for regionally restricted modulation of hyperalgesia, or the incorporation of neuron-specific promoters (e.g., Syn1 or hSyn) in AAV gene-replacement therapy to prevent overexpression toxicity, alongside strict definition of safe early-intervention time windows. Exploring downstream key effector molecules (e.g., SV2A) as PET functional imaging probes will also provide essential companion diagnostic tools for related neuropsychiatric disorders.

In summary, CELF4 serves as a cross-system molecular node connecting neural development, synaptic plasticity, chronic pain, metabolic regulation, cardiovascular remodeling, and tumor epigenetics. In-depth investigation of this protein will not only help reveal the underlying molecular networks of multisystem comorbidities but also provide potential targets for precision molecular diagnosis, rare-disease genetic counseling, and innovative targeted therapies. As single-cell multi-omics resolution continues to improve and targeted RNA therapies mature, intervention strategies directed at CELF4 are expected to overcome existing delivery and drugability barriers and achieve translation from basic mechanisms to clinical diagnostic and therapeutic tools.

## Glossary

3′UTR: 3′Untranslated Region
AAV: Adeno-Associated Virus
AH: Atypical Hyperplasia
ASD: Autism Spectrum Disorder
AUC: Area Under the Curve
BDNF: Brain-Derived Neurotrophic Factor
CELF: CUGBP Elav-like Family
CELF4: CUGBP Elav-like Family Member four
CFs: Cardiac Fibroblasts
CGRP: Calcitonin Gene-Related Peptide
CLIP-seq: Cross-Linking Immunoprecipitation Sequencing
CNS: Central Nervous System
CSDS: Chronic Social Defeat Stress
DRG: Dorsal Root Ganglia
E/I: Excitation-Inhibition
EC: Endometrial Cancer
GWAS: Genome-Wide Association Study
HUVECs: Human Umbilical Vein Endothelial Cells
IBS: Irritable Bowel Syndrome
MDD: Major Depressive Disorder
MTAG: Multi-Trait Analysis of GWAS
NDDs: Neurodevelopmental Disorders
OR: Odds Ratio
PBMCs: Peripheral Blood Mononuclear Cells
PCW: Post-Conceptional Weeks
PFC: Prefrontal Cortex
PNS: Peripheral Nervous System
qRT-PCR: Quantitative Real-Time Polymerase Chain Reaction
RBP: RNA-Binding Protein
RIP-seq: RNA Immunoprecipitation Sequencing
RRM: RNA Recognition Motif
snRNA-seq: Single-Nucleus RNA Sequencing
SNPs: Single Nucleotide Polymorphisms
SP: Subplate
TAD: Topological Associating Domain
TCGA: The Cancer Genome Atlas
TGF-β1: Transforming Growth Factor Beta 1
TRPV1: Transient Receptor Potential Cation Channel Subfamily V Member 1
UCEC: Uterine Corpus Endometrial Carcinoma
UG: Uridine-Guanine
